# Atorvastatin Exerts Antileukemia Activity via Inhibiting Mevalonate-YAP Axis in K562 and HL60 Cells

**DOI:** 10.3389/fonc.2019.01032

**Published:** 2019-10-09

**Authors:** Lei Zhang, Ting Chen, Yonghai Dou, Shaolu Zhang, Hongyan Liu, Tungalagtamir Khishignyam, Xiaofei Li, Duo Zuo, Zhe Zhang, Meihua Jin, Ran Wang, Yuling Qiu, YuXu Zhong, Dexin Kong

**Affiliations:** ^1^Tianjin Key Laboratory on Technologies Enabling Development of Clinical Therapeutics and Diagnostics, School of Pharmacy, Tianjin Medical University, Tianjin, China; ^2^State Key Laboratory of Toxicology and Medical Countermeasures, Beijing Institute of Pharmacology and Toxicology, Beijing, China; ^3^Tianjin Medical University Cancer Hospital, Tianjin, China; ^4^School of Medicine, Tianjin Tianshi College, Tianyuan University, Tianjin, China

**Keywords:** Atorvastatin, anti-leukemia, mevalonate-YAP axis, cell cycle, apoptosis

## Abstract

Novel therapeutic strategies are still urgently expected for leukemia despite undisputed success of various targeted therapeutics. The antileukemia activity of Atorvastatin, a 3-hydroxy-3-methylglutaryl coenzyme A (HMG-CoA) reductase inhibitor, on human leukemia cells was investigated. Atorvastatin inhibited K562 and HL60 cell proliferation, induced G2/M cell cycle arrest in K562 cells by down-regulating cyclinB1 and cdc2, but G0/G1 arrest in HL60 cells by up-regulating p27 and down-regulating cyclinD1 and p-pRb. Atorvastatin also induced apoptosis in both cell lines, in which the reactive oxygen species (ROS)-related mitochondrial apoptotic signaling might be involved, with increase of ROS and Bax/Bcl-2 ratio, loss of mitochondrial membrane potential (MMP), release of cytochrome C into cytosol, and activation of Bax/Caspase-9/Caspase-3/PARP pathway. Inhibition of YAP nuclear localization and activation by Atorvastatin was reversed by the addition of mevalonate, GGPP, or FPP. Further, the effects on cell cycle arrest- and apoptosis- related proteins by Atorvastatin were alleviated by addition of mevalonate, suggesting the antileukemia effect of Atorvastatin might be through mevalonate-YAP axis in K562 and HL60 cells. Our results suggest that Atorvastatin might be used for leukemia therapy while evidence of clinical efficacy is required.

## Introduction

Leukemia is a group of hematopoietic malignancies with high mortality and morbidity (1). It can be either acute or chronic depending on the rate of disease progression. Chronic myeloid leukemia (CML) and acute myeloid leukemia (AML) are main sub-type of leukemia of myeloid origin (2). CML is caused by oncogene BCR-ABL. BCR-ABL protein exhibits constitutive tyrosine kinase activity and plays a central role in the pathogenesis of CML (3). AML is molecularly and clinically heterogeneous, caused by many genetic and epigenetic aberrations (4). Genetic mutations in FLT3, one of the widely identified genes associated with AML pathogenesis, account for at least 30% of AML (5). FLT3 encodes the Fms-like tyrosine kinase-3 which is a class III receptor tyrosine kinase and closely involved in cell growth and differentiation. Molecular-targeted drugs, such as BCR-ABL inhibitors for CML (imatinib, nilotinib) and FLT3 inhibitors for AML (sorafenib and midostaurin), have made a great success in therapy of leukemia in the past decades (5–7). Unfortunately, the insufficient response to molecular-targeted drugs and the acquired drug resistance remain major therapeutic challenges (7–9). Therefore, novel therapies for leukemia are still needed.

The mevalonate (MVA) pathway has been widely known for its role in isoprenoid biosynthesis in mammals. The substrate, 3-hydroxy-3-methyl-glutaryl-CoA (HMG-CoA), is conversed to MVA by 3-hydroxy-3-methyl-glutaryl-CoA reductase (HMGCR). MVA can be metabolized to farnesyl pyrophosphate (FPP), geranylgeranyl pyrophosphate (GGPP) or other biomolecules. Among these metabolites, FPP and GGPP are two key factors controlling post-translational prenylation of the small GTPases (Ras, Rac, Rho, etc.). The GTPases subsequently participate in various cellular responses (10, 11). Recently, the MVA pathway has become a pivotal therapeutic target in cancer treatment (12, 13). Targeting MVA pathway is expected to inhibit the biosynthesis of isoprenoids including FPP and GGPP, block the post-translational prenylation of the small GTPases, and finally inhibit cell survival and proliferation (12).

As HMGCR inhibitors, statins have attracted much attention for development as anti-cancer drug candidates (14). Blockade of MVA pathway by statins inhibits the downstream FPP and GGPP which in turn modifies the localization and activity of small GTPases, and therefore blocks corresponding cellular signaling (15). Numerous studies have demonstrated that statins exhibit pleiotropic effects beyond the well-known lipid-lowering action, such as cancer prevention, anti-inflammation, and immune regulation (14).

In the present research, we investigated the underlying mechanism involved in anti-leukemia activity of Atorvastatin on CML K562 cells and AML HL60 cells.

## Materials and Methods

### Reagents and Antibodies

Atorvastatin calcium, Mevalonic acid, GGPP, FPP, Propidium iodide (PI) and 2′,7′-dichlorofluorescein diacetate (DCFH-DA) were purchased from Sigma-Aldrich (St. Louis, MO, USA). 3-(4,5-Dimethyl-2-thiazolyl)-2,5-diphenyl-2H-tetrazolium bromide (MTT) was from Amresco (Solon, OH, USA). 5,5′,6,6′-tetrachloro-1, 1′,3,3′-tetraethylbenzimidazole-carbocyanide iodine (JC-1) and cell mitochondria isolation kit were obtained from Beyotime Biotechnology (Shanghai, China). Annexin V-FITC Apoptosis Detection Kit, as well as antibodies against p-pRb (S780) (1:1,000), p27 (1:1,000) and cyclinD1 (1:1,000) were from BD Biosciences Pharmingen (San Jose, CA, USA). Antibodies against pRb (1:1,000), cyclinB1 (1:2,000), cdc2 (1:1,000), caspase-3 (1:1,000), caspase-9 (1:1,000), poly (ADP-ribose) polymerase (PARP) (1:1,000), Cytochrome c (1:1,000), YAP (1:1,000), p-YAP (Ser127) (1:1,000), Rho A (1:1,000), β-actin (1:1,000), anti-mouse (1:2,000), anti-rabbit HRP-conjugated and anti-rabbit Alexa Fluor 488-conjugated secondary antibodies (1:2,000) were from Cell Signaling Technology (Danvers, MA, USA). Antibodies against Bcl-2 (1:1,000), Bax (1:1,000) and Lamin B (1:500) were from Santa Cruz Biotechnology (Santa Cruz, CA, USA).

### Cell Lines and Primary Cultures

The human CML K562 and human AML HL60 cell lines were purchased from Cell Resource Center, Peking Union Medical College (Beijing, China). Cells were cultured under 5% CO_2_ at 37°C in RPMI 1640 medium supplemented with fetal bovine serum (FBS, 10%), penicillin (100 U/ml) and streptomycin (100 μg/ml) (Biological Industries, Beit Haemek, Israel). Three human blood samples were collected from healthy volunteers with informed consent in accordance with the approval of the Ethic Committee of Tianjin Medical University. As we previously reported (16), the peripheral blood mononuclear cells (PBMCs) were isolated from total blood samples by using Human Lymphocyte Separation Medium (Dakewe, Beijing, China) and maintained in RPMI medium containing 20% (v/v) FBS at 37°C in a 5% CO_2_.

### MTT Assay

Cell proliferation was assessed by MTT assay, as described in our previously report (17). Briefly, cell suspension (200 μl, 3 × 10^4^ cells/ml) of K562, HL60, or PBMCs was seeded into a 96-well plate and exposed to various concentrations of Atorvastatin (0, 0.3125, 0.625, 1.25, 2.5, 5, 10, 20, 40, and 80 μM) for 48 h at 37°C. Then, the respective cells were further incubated with MTT (5 mg/ml). After 4 h, the absorbance at 490 nm was measured using a microplate reader iMark (BIO-RAD, Hercules, CA, USA). The IC_50_ values were calculated by use of GraphPad Prism 5 software (GraphPad Software, San Diego, CA, USA) according to the logistic curve.

### Cell Cycle Analysis

The effect of Atorvastatin on cell cycle progression was determined using PI staining (18). After treated with Atorvastatin (0, 5, 10, and 20 μM) for 48 h, cells (4 × 10^5^ cells/ml) were harvested, centrifuged and washed twice with phosphate-buffered saline (PBS) before fixation with 70% ethanol overnight at 4°C. Then, the fixed cells were centrifuged, stained with PI solution (50 μg/ml PI, 100 μg/ml RNase A and 0.5% Triton X-100) for 30 min at 4°C in the dark. The stained cells were further subjected to flow cytometer BD Accuri C6 (BD Biosciences, San Jose, CA, USA) for analysis.

### Apoptosis Assay

For the apoptosis analysis, Annexin V-FITC/PI double staining was used as reported by us before (17, 19). The cells (4 × 10^5^ cells/ml) were exposed to various concentrations of Atorvastatin (0, 5, 10, and 20 μM) for 48 h at 37°C. Then the treated cells were collected and double stained with Annexin V-FITC/PI solution (100 μl of binding buffer containing Annexin V-FITC and PI) for 15 min in the dark at room temperature. The cells were then resuspended in 200 μl binding buffer and immediately analyzed by a FACS Verse flow cytometer (BD Biosciences, San Jose, CA, USA). The number of viable (AnnexinV^−^/PI^−^), early apoptotic (AnnexinV^+^/PI^−^) and late apoptotic (AnnexinV^+^/PI^+^) cells were quantified, respectively, using Flow Jo Software (Tristar, CA, USA).

### Determination of Reactive Oxygen Species (ROS)

Intracellular ROS levels were determined as we reported previously (20, 21). The cells were treated with different concentrations of Atorvastatin (0, 5, 10, and 20 μM) for 24 h, and stained with DCFH-DA (10 μM) for 30 min. Thereafter, cells were harvested, washed, and resuspended in PBS followed by examination on a FACS Verse flow cytometer (BD Biosciences, San Jose, CA, USA) (λ_ex_: 488 nm, λ_em_: 530 nm).

### Measurement of Mitochondrial Membrane Potential (MMP)

MMP was assessed using flow cytometry after staining with JC-1, a dual-emission membrane potential-sensitive probe, as we reported previously (22). The cells (4 × 10^5^ cells/ml) were incubated with different concentrations of Atorvastatin (0, 5, 10, and 20 μM) in 6-well plates for 24 h at 37°C. Subsequently, the cells were washed, resuspended in PBS and stained with JC-1 (2 μM) for 20 min at 37°C. Finally, the resulting fluorescent intensity (λ_em_: 530 nm for the monomeric JC-1, λ_em_: 590 nm for the aggregate of JC-1) was measured by using flow cytometer FACS Verse. The fluorescence intensity ratio of aggregates over monomers was calculated as MMP. Data were analyzed by Flow Jo Software (Tristar, CA, USA).

### Preparation of Cytosolic Fraction and Mitochondrial Fraction

The cells (4 × 10^5^ cells/ml) were grown in the presence of different concentrations of Atorvastatin (0, 5, 10, and 20 μM) in 6-well plates for 24 h at 37°C. After incubation, the cells were washed with PBS, lysed in fresh mitochondria isolation buffer containing 1 mM of phenyl methyl sulfonyl fluoride (PMSF) and homogenized on ice by a Dounce-type glass homogenizer. The homogenate was centrifuged at 1,000 g for 5 min at 4°C and the supernatant was collected and further centrifuged at 12,000 g for 10 min at 4°C, with the resulting supernatants as cytosolic fraction and the pellet as crude mitochondria. Subsequently, the crude mitochondria pellet was lysed in mitochondria lysis buffer containing 1 mM PMSF. After centrifugation at 12,000 g for 10 min at 4°C, the supernatant was finally collected as mitochondrial fraction.

### Western Blot

Western blot analysis was performed as previously described (23, 24). The cells (4 × 10^5^ cells/ml) were treated with different concentrations of Atorvastatin (0, 5, 10, and 20 μM) for 48 h at 37°C. To extract the total and nuclear proteins, the cells were harvested, lysed using RIPA lysis buffer and NE-PER Nuclear and Cytoplasmic Extraction kit (Thermo Fisher Scientific, Waltham, MA, USA), respectively. Then, the protein quantification was conducted using BCA protein Assay Kit. Equal amounts of protein (30–60 μg) resuspended and denatured in SDS sample buffer were subjected to 10% sodium dodecyl sulfate polyacrylamide gel electrophoresis (SDS-PAGE) and transferred onto polyvinylidene fluoride (PVDF) membranes (Millipore, Billerica, MA, USA). After 1 hour-blocking in 5% non-fat milk, the membranes were exposed to specified primary antibodies in Tris-buffered saline (TBS-T; 25-mM Tris-HCl (pH 7.6), 150-mM NaCl, and 0.05% Tween 20) containing 5% BSA, followed by the secondary antibodies at room temperature for 1 h in TBS-T containing 5% BSA. Finally, the blots were visualized on a Bio-Rad ChemiDocTM XRS+ System using enhanced chemiluminescence (ECL) reagents and quantified with Image LabTM Software.

### Immunofluorescence Staining

YAP localization was detected by immunofluorescence staining (25). Briefly, the treated cells were fixed in 4% paraformaldehyde for 10 min at room temperature, rinsed with PBS, and permeabilized with 0.2% Triton X-100 for 10 min. Then the fixed cells were blocked in PBS containing 3% BSA and 3% FBS for 30 min at 37°C. Thereafter, cells were further incubated with rabbit anti-YAP antibody for 1 h at 37°C followed by Alexa Fluor 488-conjugated secondary antibody for 1 h at 37°C. Then, the nuclei were counterstained with Hoechst. Finally, the expression of YAP was visualized under Olympus FV1000 laser scanning confocal microscope (Olympus, Japan). Images were acquired by using FV10-ASW3.0 software. YAP nuclear localization was examined via predominant nuclear staining of YAP in cells for each experiment.

### Statistical Analysis

Data are presented as mean ± standard deviation (SD) from at least three independent experiments. All values of *P* were analyzed using the Student's *t*-tests with GraphPad Prism 5 software (GraphPad, San Diego, CA, USA). *P* < 0.05 was considered as statistically significant.

## Results

### Atorvastatin Inhibits Proliferation of Leukemia Cells With Low Toxicity on Normal PBMCs

**Figure 1:**
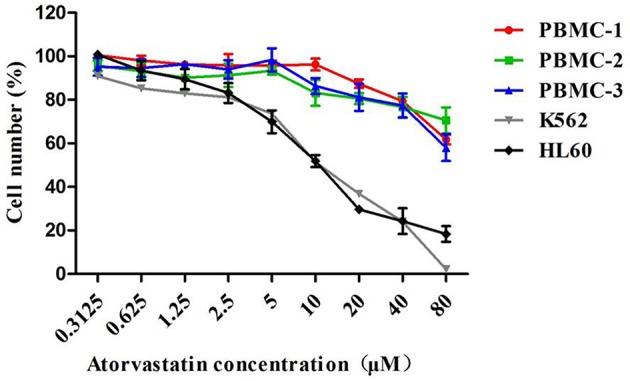
Atorvastatin inhibits proliferation of leukemia cells with low toxicity on normal PBMCs. K562, HL60, and normal PBMCs were incubated with Atorvastatin (0, 0.3125, 0.625, 1.25, 2.5, 5, 10, 20, 40, and 80 μM) for 48 h. Cell viability was determined by MTT assay. Data are presented as mean ± SD of three independent experiments conducted in triplicate.

The effect of Atorvastatin on the growth of CML K562, AML HL60, as well as normal PBMCs was investigated by MTT assay. As shown in Figure 1, Atorvastatin showed similar growth inhibition potency on K562 and HL60 cells. The IC_50_ values (half-maximal inhibitory concentration) were calculated to be 10.55 μM for K562 and 10.26 μM for HL60. However, even after treatment with 80 μM of Atorvastatin, <50% inhibition of PBMCs was indicated, suggesting the weak cytotoxicity of Atorvastatin on normal cells.

### Atorvastatin Induces Cell Cycle Arrest in K562 and HL60 Cells

**Figure 2:**
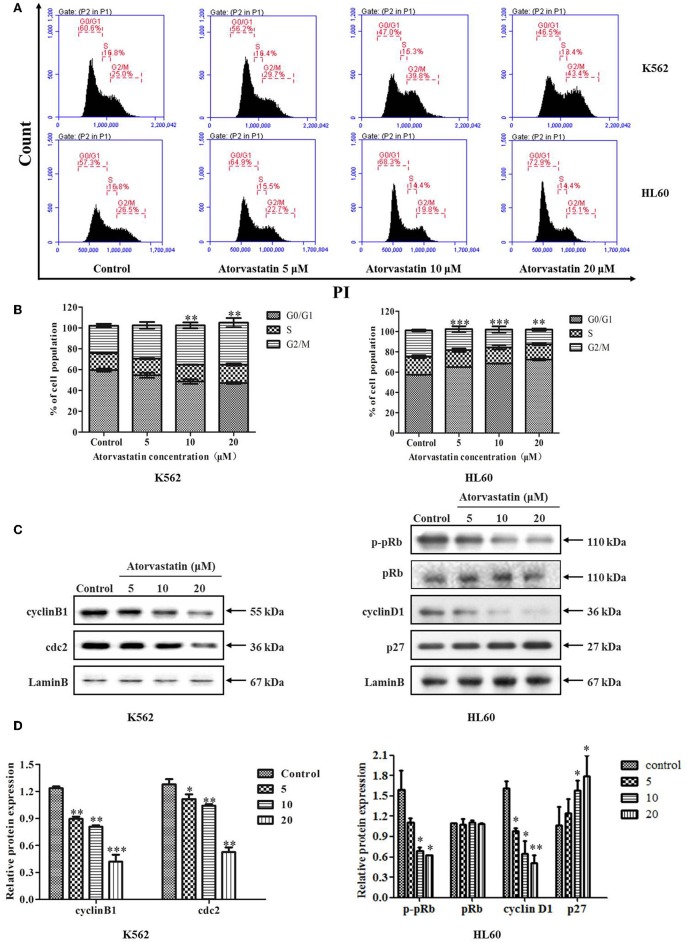
Atorvastatin induces cell cycle arrest in K562 and HL60 cells. K562 and HL60 cells were incubated with Atorvastatin (0, 5, 10, and 20 μM) for 48 h. **(A)** Cell cycle distribution was analyzed by flow cytometer, and the representative images were shown. **(B)** The percentages of total cells at G0/G1, S, and G2/M phases in K562 and HL60 cells were shown and statistically analyzed. **(C)** The levels of cyclinB1 and cdc2 in K562 cells, as well as cyclinD1, p27, p-pRb, and pRb in HL60 cells were determined by western blot. **(D)** Bar graphs show the relative levels of cyclinB1, cdc2, cyclinD1, p27, p-pRb, and pRb. Data are presented as mean ± SD of three independent experiments. *p < 0.05, **p < 0.01, ***p < 0.001 vs. control.

To investigate whether the cell cycle progression was affected by Atorvastatin, we analyzed the cell cycle distribution of K562 and HL60 cells after Atorvastatin treatment. As illustrated in Figures 2A,B, the population of K562 cells in G2/M phase increased dose-dependently, whereas that of HL60 cells in G0/G1 phase increased. These results suggested that Atorvastatin delayed cell cycle progression by inducing G2/M arrest in K562 cells and G0/G1 arrest in HL60 cells.

The cell cycle checkpoint proteins play an essential role in regulating cell cycle progression. To investigate the molecular mechanism involved in Atorvastatin-mediated cell cycle arrest in both cell lines, G2/M regulatory proteins such as cyclinB1 and cdc2 in K562 cells, as well as the key regulators of G1 to S phase transition such as cyclinD1, p27, and the downstream p-pRb in HL60 cells were analyzed by western blot. As shown in Figures 2C,D, following Atorvastatin treatment, the levels of cyclin B1 and cdc2 were significantly reduced in K562 cells dose-dependently. In the case of HL60 cells, there was a significant reduction of cyclin D1 and p-pRb along with an obvious enhancement of p27 by Atorvastatin treatment in comparison with control. There is no significant change in pRb expression in HL60 cells.

### Atorvastatin Induces Mitochondria-Dependent Apoptosis in K562 and HL60 Cells

**Figure 3:**
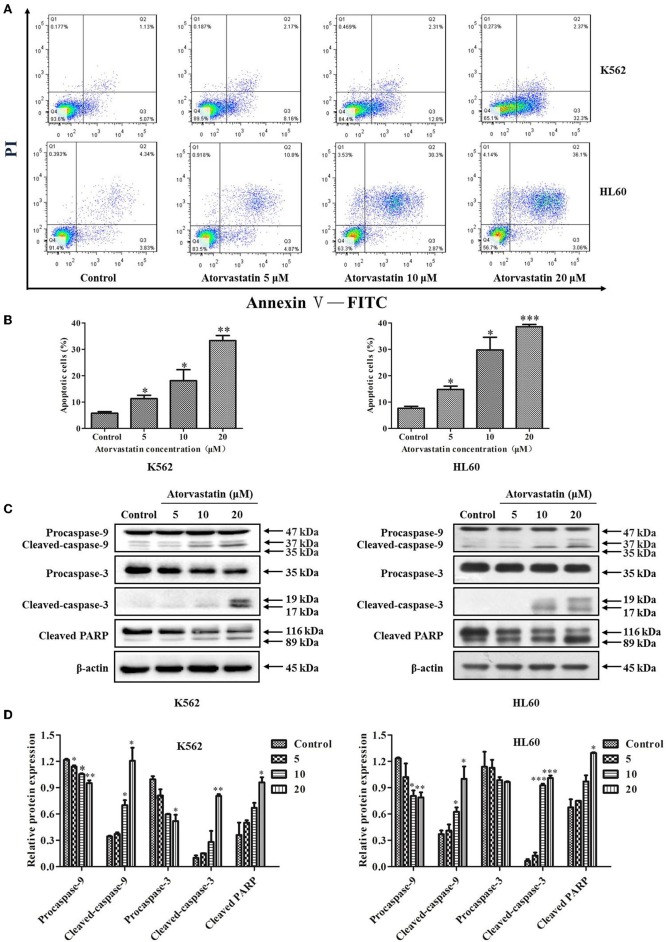
Atorvastatin induces apoptosis in K562 and HL60 cells. K562 and HL60 cells were incubated with Atorvastatin (0, 5, 10, and 20 μM) for 48 h. **(A)** The apoptosis was examined by flow cytometer, and the representative images were shown. **(B)** The percentages of apoptotic cells were shown and statistically analyzed. **(C)** The levels of caspase-9, caspase-3, and PARP were determined by western blot. **(D)** Bar graphs show the relative levels of caspase-9, caspase-3, and PARP. Data are presented as mean ± SD of three independent experiments. *p < 0.05, **p < 0.01, ***p < 0.001 vs. control.

To further decipher Atorvastatin-induced cytotoxicity, FITC-conjugated Annexin V and PI double staining was performed in Atorvastatin-treated leukemia cells. As indicated in Figures 3A,B, there was a dramatic increase in early (Annexin V^+^/PI^−^) and late (Annexin V^+^/PI^+^) apoptotic cell population of K562 and HL60 cells, respectively. In the control group, Annexin V-labeled population (early apoptotic for K562) and both PI- and Annexin V-labeled population (late apoptotic for HL60) were 5.07 and 4.34%, while those in the Atorvastatin-exposed (20 μM) group were 32.3% (for K562) and 36.1% (for HL60), respectively. The levels of apoptosis-related proteins were determined. As shown in Figures 3C,D, Atorvastatin treatment resulted in a pronounced increase of cleaved caspase-9, caspase-3, and PARP in both cell lines.

**Figure 4:**
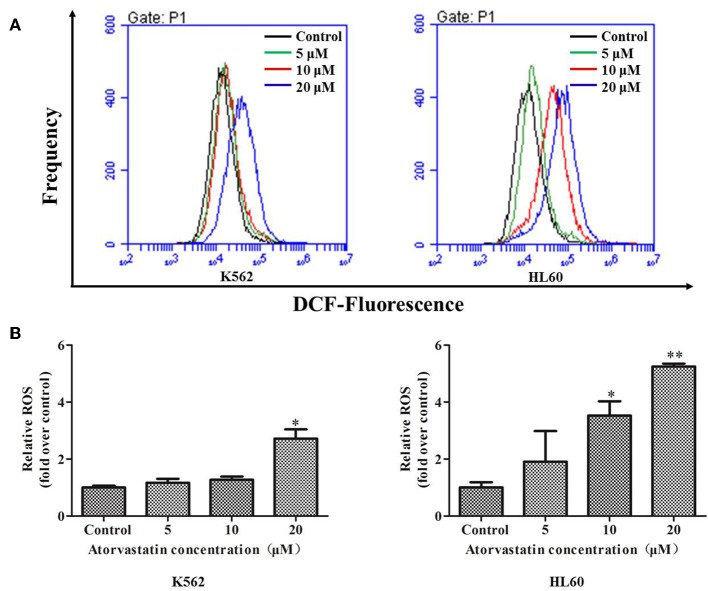
Atorvastatin induces intracellular ROS generation in K562 and HL60 cells. K562 and HL60 cells were incubated with Atorvastatin (0, 5, 10, and 20 μM) for 24 h. **(A)** Intracellular ROS levels were detected by flow cytometer, and the representative images were shown. **(B)** The percentages of relative ROS were shown and statistically analyzed. Data are presented as mean ± SD of three independent experiments. *p < 0.05, **p < 0.01 vs. control.

It has been proven that the excessive ROS generation can induce mitochondrial depolarization and dysfunction, which subsequently result in programmed cell death including apoptosis (26). To elucidate whether ROS generation is involved in Atorvastatin-induced apoptosis, K562 and HL60 cells were treated with Atorvastatin (5, 10, and 20 μM) and the intracellular ROS levels were quantified by flow cytometer. Figures 4A,B showed that the generated ROS increased markedly in both cell lines after Atorvastatin treatment. These data demonstrated that the Atorvastatin-induced apoptosis might be related to ROS generation in K562 and HL60 cells.

**Figure 5:**
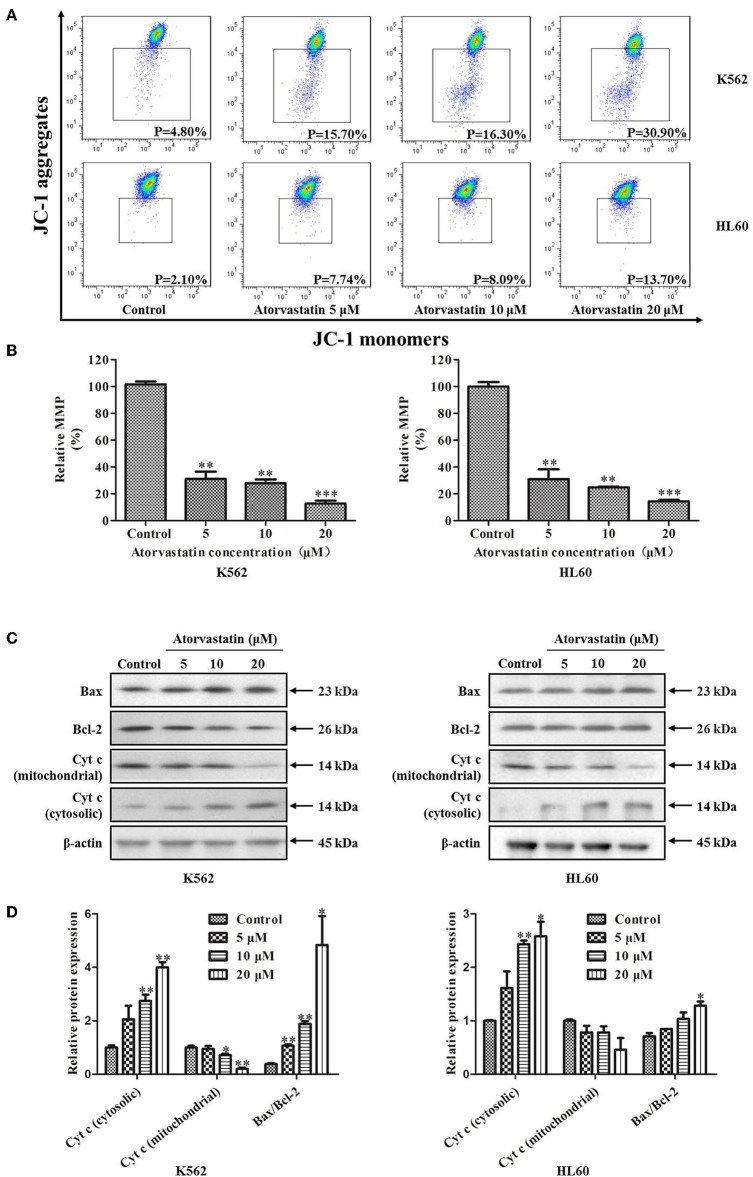
Atorvastatin induces loss of MMP in K562 and HL60 cells. K562 and HL60 cells were incubated with Atorvastatin (0, 5, 10, and 20 μM) for 24 h. **(A)** MMP was examined by flow cytometer and the data were analyzed using Flow Jo Software. The representative images were shown. **(B)** The percentages of relative MMP were shown and statistically analyzed. **(C)** The levels of Bcl-2, Bax, and Cytochrome c (Cyt-c) were determined by western blot. **(D)** Bar graphs show the relative levels of Bcl-2, Bax, and Cytochrome *c*. Data are presented as mean ± SD of three independent experiments. *p < 0.05, **p < 0.01, ***p < 0.001 vs. control.

To determine whether Atorvastatin-induced leukemia cell apoptosis is mitochondria-dependent or not, the loss of MMP and the expressions of several apoptotic regulators were examined in cells with or without Atorvastatin treatment. As shown in Figure 5A, percentage of cells with MMP loss after 24 h-Atorvastatin-treatment (20 μM) increased by 26.10 and 11.60% for K562 and HL60 cells, respectively. Consistently, the relative MMP of cells treated by Atorvastatin reduced in a dose dependent manner in both K562 and HL60 cells (Figure 5B). The expressions of Bcl-2, Bax and Cytochrome *c* were also determined. As shown in Figures 5C,D, Atorvastatin markedly increased Bax and reduced Bcl-2, leading to an enhancement of Bax/Bcl-2 ratio. Moreover, the release of Cytochrome c from mitochondria was also observed in response to Atorvastatin exposure. Collectively, these findings indicated that Atorvastatin induced ROS-mediated mitochondria-dependent apoptosis in K562 and HL60 cells.

### Atorvastatin Inhibits YAP Nuclear Localization in K562 and HL60 Cells

**Figure 6:**
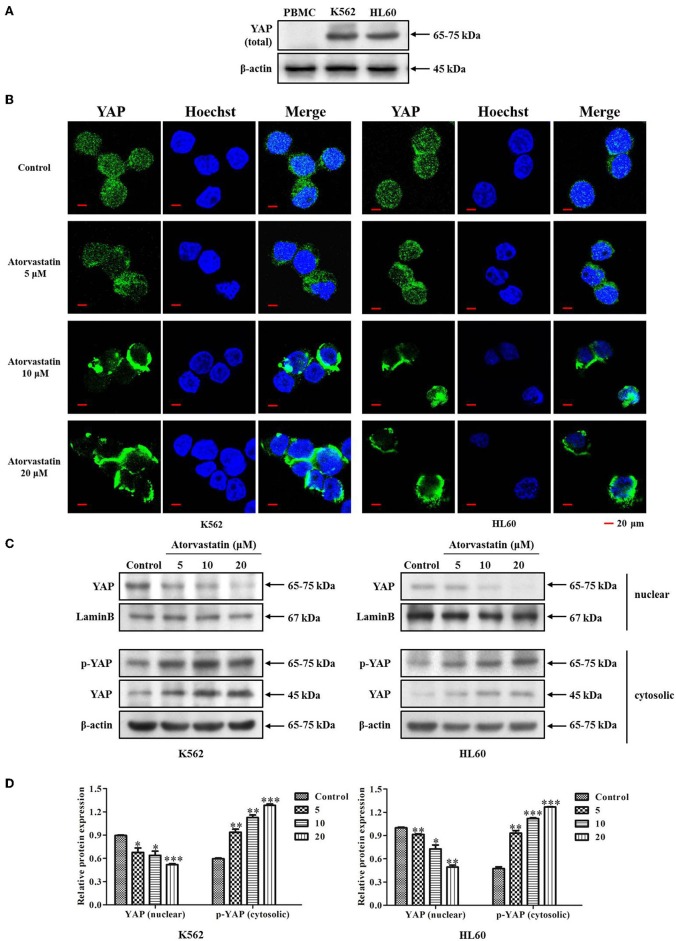
Atorvastatin inhibits YAP nuclear localization in K562 and HL60 cells. **(A)** The expression of YAP in leukemia cell lines (K562 and HL60) and PBMCs from healthy volunteers (*n* = 3) was determined by western blot analysis. **(B)** K562 and HL60 cells were incubated with Atorvastatin (0, 5, 10, and 20 μM) for 48 h. The subcellular localization of YAP was examined by immunofluorescence and visualized under Olympus laser scanning confocal microscope. The nuclei were counterstained with Hoechst. The representative images were shown. Scale bar, 20 μm. **(C)** The levels of YAP in nucleus and p-YAP (Ser127) in cytoplasm were determined by western blot. **(D)** Bar graphs show the relative levels of YAP and p-YAP (Ser127). Data are presented as mean ± SD of three independent experiments. *p < 0.05, **p < 0.01, ***p < 0.001 vs. control.

It has been reported that YAP acts as an oncoprotein in many solid cancers, and plays important roles in regulating stemness of cancer cells (27, 28). Aiming to identify the potential role of YAP in leukemia cells, we firstly examined the expression of YAP in leukemia cell lines (K562 and HL60) and PBMCs from healthy volunteers (*n* = 3). Figure 6A showed that YAP expression level was significantly higher in K562 and HL60 cells compared with that in PBMC. Our finding supports that YAP might be a potential biomarker of leukemia.

YAP cytoplasmic retention is known as a signal of YAP inhibition (28, 29). Previous reports indicated that statins suppress YAP activity and nuclear localization in tumor cells, thus exhibiting anticancer effect (15, 30). To determine whether Atorvastatin possesses similar effect in regulating YAP nuclear localization in K562 and HL60 cells, we then examined the subcellular localization of YAP in response to Atorvastatin treatment by immunofluorescence and western blot analysis. As expected, a robust YAP nuclear localization was observed in both cell lines without drug treatment. However, after exposure to Atorvastatin, a significant cytoplasmic relocalization of YAP occurred in K562 and HL60 cells (Figure 6B). This result was confirmed by western blot. As shown in Figures 6C,D, Atorvastatin induced an increase of YAP phosphorylation (Ser127) in the cytosolic fraction and a reduction of YAP expression in the nuclear fraction. Taken together, these results suggested Atorvastatin inhibited nuclear localization of YAP in K562 and HL60 cells.

### Inactivation of YAP by Atorvastatin Is Mediated by MVA Cascade

**Figure 7:**
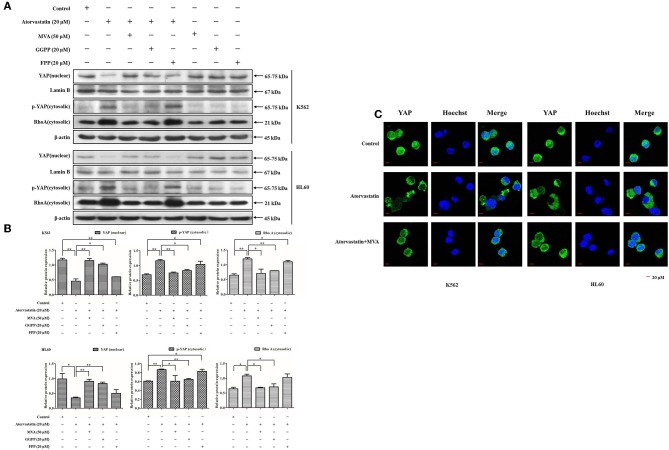
Inactivation of YAP by Atorvastatin is mediated by MVA cascade. K562 and HL60 cells were incubated with Atorvastatin (0 and 20 μM) in presence or absence of exogenous MVA (50 μM), FPP (20 μM), or GGPP (20 μM) for 48 h. **(A)** The levels of YAP in nucleus, p-YAP (Ser127) and RhoA in cytosol were examined by western blot. **(B)** Bar graphs show the relative levels of YAP, p-YAP (Ser127), and RhoA. **(C)** The subcellular localization of YAP was examined by using immunofluorescence and visualized under Olympus laser scanning confocal microscope. The nuclei were counterstained with Hoechst. The representative images were shown. Scale bar, 20 μm. Data are presented as mean ± SD of three independent experiments. *p < 0.05, **p < 0.01 vs. control.

YAP activity is reported to be regulated by MVA cascade apart from Hippo signaling (30, 31). To gain insights into the molecular mechanism of Atorvastatin-induced YAP inactivation in leukemia cells, we then examined the effect of Atorvastatin on YAP cytoplasmic relocalization in the presence or the absence of exogenous MVA, FPP, or GGPP. As shown in Figures 7A,B, 20 μM of Atorvastatin obviously enhanced cytosolic p-YAP (Ser127) and decreased nuclear YAP in both cell lines. These inhibitory effects were completely rescued by MVA co-treatment and partially rescued by GGPP and FPP co-treatment. RhoA is an important effector of GGylation for signaling to YAP (25). In this research, the increase in cytosolic level of RhoA by Atorvastatin was fully reversed by MVA or GGPP co-treatment, and partially reversed by FPP co-treatment, suggesting that RhoA was involved in Atorvastatin-induced YAP inactivation. Furthermore, Figure 7C showed that addition of MVA reversed YAP cytoplasmic redistribution induced by Atorvastatin in K562 and HL60 cells. Collectively, our research suggested that inactivation of YAP by Atorvastatin is mediated by MVA cascade.

### Atorvastatin Exerts Anti-leukemia Activity via MVA-YAP Axis in K562 and HL60 Cells

**Figure 8:**
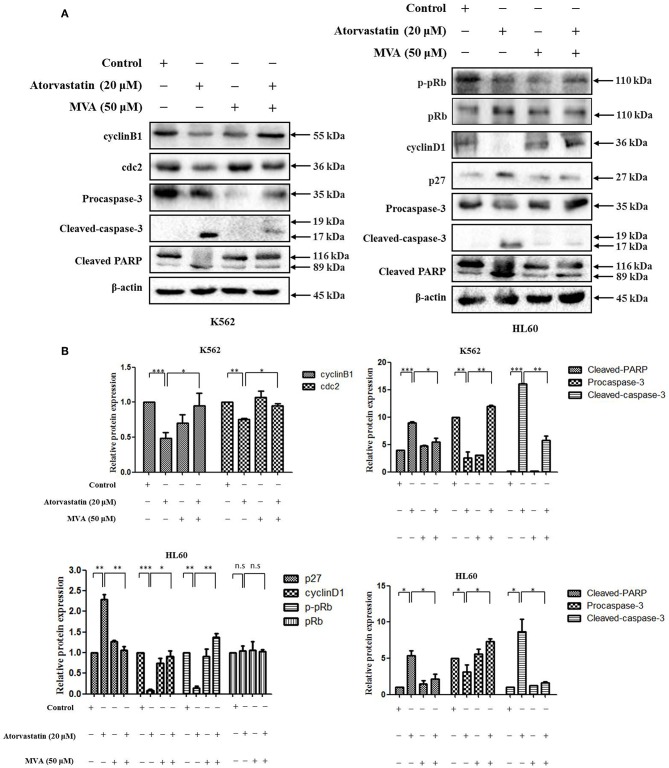
Atorvastatin exerts antileukemia activity via MVA-YAP axis in K562 and HL60 cells. K562 and HL60 cells were incubated with Atorvastatin (0 and 20 μM) in presence or absence of exogenous MVA (50 μM) for 48 h. **(A)** The levels of cyclinB1 and cdc2 in K562 cells, as well as cyclinD1, p27, p-pRb, and pRb in HL60 cells, caspase-3 and PARP in both cell lines were determined by western blot. **(B)** Bar graphs show the relative levels of cyclinB1, cdc2, cyclinD1, p27, p-pRb, pRb, caspase-3, and PARP, Data are presented as mean ± SD of three independent experiments. *p < 0.05, **p < 0.01, ***p < 0.001 vs. control. n.s., not significant.

To further examine the signaling mechanism for anti-leukemia effect of Atorvastatin on K562 and HL60 cells, the cells were treated with Atorvastatin in presence or absence of exogenous MVA, and the levels of cell cycle- and apoptosis- related effectors were determined by western blot. As shown in Figure 8, the decrease of cyclin B1 and cdc2 in response to Atorvastatin was reversed by MVA in K562 cells. And in HL60 cells, addition of MVA suppressed the effect of Atorvastatin on cyclin D1, p-pRb, and p27 levels. The expression of pRb was stable in HL60 cells. In addition, the effect of Atorvastatin on cleaved caspase-3 and PARP was also rescued by MVA in both cell lines. Our result suggests that Atorvastatin exerts anti-leukemia activity via MVA-YAP axis in K562 and HL60 cells.

## Discussion

Atorvastatin is a member of statins which lower cholesterol through inhibiting the HMG-CoA reductase of the mevalonate pathway for cholesterol biosynthesis. Accumulating evidences have identified that mevalonate cascade dysregulation may possess sufficient oncogenic potential, therefore conferring statins potent anticancer effect (11, 12). And a surge of publications in the past decade have reported that statins exhibit anti-proliferative and pro-apoptotic effects in many types of cancers (14, 15). However, the in-depth mechanism of anti-leukemic effect of Atorvastatin remains to be elucidated. Herein, we have demonstrated that Atorvastatin exhibited anti-leukemia via inhibiting MVA-YAP axis, subsequently leading to proliferation suppression and apoptosis induction in CML K562 and AML HL60 cells.

Our research has confirmed that Atorvastatin dose-dependently inhibited proliferation of K562 and HL60 cells but showed low cytotoxicity on normal PBMCs. Atorvastatin induced cell cycle arrest in K562 and HL60 cell lines. In K562 cells, G2/M cell cycle arrest with cyclinB1 and cdc2 downregulation was observed, while in HL60 cells, G0/G1 arrest with a reduction of cyclin D1, and p-pRb, as well as p27 enhancement occurred. This difference might be attributed to different genetic profiles of K562 and HL60 cell lines. Moreover, Atorvastatin significantly promoted mitochondria-dependent apoptosis in both cell lines. Taken together, these data suggest that Atorvastatin exhibits anti-leukemia activity mainly through cell cycle arrest and apoptosis-induction.

YAP is a core component of the Hippo pathway controlling tissue proliferation, organ size and stem cell self-renewal (27, 28). Numerous studies have revealed that YAP is involved in the pathogenesis of various malignancies and might be a potential target for cancer therapy (29). In our study, to explore the potential role of YAP in leukemia, we investigated the expression of YAP in 2 leukemia cell lines (K562 and HL60) and PBMCs from healthy volunteers. Our results showed that YAP was significantly up-regulated in K562 and HL60 cells compared with PBMC, suggesting that YAP might be a biomarker of leukemia.

Hippo pathway is a well-known tumor suppressor cascade (28, 32). YAP is known to locate downstream of Hippo pathway (28, 31). However, because of the negative regulation of YAP by Hippo kinases and the inactive status of Hippo pathway in various cancers, efforts of drug discovery targeting Hippo cascade are frustrated (30, 33). Fortunately, MVA-YAP axis is thought to be a new trial (30). The MVA cascade intermediates, GGPP and FPP, participate in prenylation (either farnesylation or GGylation) of the intracellular small GTPase (11). Notably, RhoA, a Rho-GTPase closely related with malignancy, is activated by GGPP via GGylation and membrane anchoring, which in turn positively regulate YAP activity (30, 34). With the developed recognition of oncogenic YAP and its biological functions intersecting with the MVA cascade, some new drug candidates targeting YAP directly or indirectly have been found (29, 35). Statins are exactly one kind of these drug candidates. Statins deplete cellular FPP and GGPP by decreasing the levels of mevalonate, thereby block prenylation of downstream effectors and regulate cellular activities.

In our research, Atorvastatin suppressed YAP activity and nuclear localization in K562 and HL60 cells. This effect was reversed by the addition of exogenous MVA, FPP, or GGPP. The inactivation of Rho A, a redistribution from membrane to a diffused cytosolic location, by Atorvastatin could also be rescued by exogenous MVA, FPP, or GGPP, suggesting that Atorvastatin inhibited YAP nuclear localization and activity in a mevalonate-dependent manner in K562 and HL60 cells. Moreover, treatment with MVA relieved the effects on cell cycle regulators and apoptosis-related molecules by Atorvastatin in both cell lines, further suggesting the anti-leukemia effect of Atorvastatin on K562 and HL60 cells was through MVA-YAP axis.

It is noteworthy that the doses of Atorvastatin used in this research largely exceed those used for cardiovascular disease therapy (28). However, considering its prominent antileukemia effect and low toxicity, further investigation including drug combination with other leukemia therapeutics with reduced dosage, deserves to be carried out.

In summary, the HMG-CoA reductase inhibitor Atorvastatin induced cell cycle arrest, intrinsic mitochondria-dependent apoptosis in CML K562 and AML HL60 cells, in which the MVA-YAP axis might be involved. Our findings reveal that administration of atorvastatin clinically may be beneficial for leukemia therapy in the future.

## Data Availability Statement

The raw data supporting the conclusions of this manuscript will be made available by the authors, without undue reservation, to any qualified researcher.

## Author Contributions

This study was designed and conceived by DK, YZ, and YQ. Experiments were performed by LZ, TC, YD, SZ, HL, TK, and XL. DZ, ZZ, MJ, and RW performed data analysis. LZ and YQ wrote the manuscript. DK revised the manuscript. All authors read and approved the final version of the manuscript.

### Conflict of Interest

The authors declare that the research was conducted in the absence of any commercial or financial relationships that could be construed as a potential conflict of interest.
